# Omics of Long-Lived Mammals and Links to Human Centenarians

**DOI:** 10.1093/geroni/igaa057.3128

**Published:** 2020-12-16

**Authors:** Gregory Tombline, Jonathan Gigas, Matthew Simon, Yousin Suh, Andrei Seluanov, Vera Gorbunova

**Affiliations:** 1 University of Rochester, Rochester, New York, United States; 2 Columbia University, New York, New York, United States

## Abstract

Mammalian species differ up to 100-fold in their aging rates and maximum lifespans. Long-lived mammals appear to possess traits that extend lifespan and healthspan. Pro-longevity mechanisms are complex traits afforded by connections between metabolism and protein functions that are unlikely to be predicted by genomic approaches alone. Thus, metabolomics and proteomics studies are required to understand the mechanisms of longevity. Sirtuin 6 will be presented as an example of a protein that evolved enhanced enzymatic function in long-lived species and also demonstrates enhanced activity and unique alleles in human centenarians. Proteome analysis reveal several longevity related proteins such as Cip1/p21, FOXO3, TOP2A, AKT1, RICTOR, INSR and SIRT6 harboring PTM sites that preferentially appear in either short- or long-lived species. The prospects of enhancing life expectancy and healthspan of humans by altering metabolism and proteoforms with drugs that mimic changes observed in long-lived species will be discussed.

